# 4-(2-Chloro­ethyl)morpholinium picrate

**DOI:** 10.1107/S1600536809035405

**Published:** 2009-09-12

**Authors:** Rajni Kant, Sabeta Kohli, Lovely Sarmal, B. Narayana, S. Samshuddin

**Affiliations:** a Department of Physics, University of Jammu, Jammu Tawi 180 006, India; b Department of Studies in Chemistry, Mangalore University, Magalagangotri 574 199, India

## Abstract

The title compound, C_6_H_13_ClNO^+^·C_6_H_2_N_3_O_7_^−^, was synthesized from picric acid and 4-(2-chloro­ethyl)morpholine. The crystal structure is stabilized by C—H⋯O and N—H⋯O hydrogen-bond inter­actions.

## Related literature

For the homeopathic uses of the metal derivatives of picric acid, see: Maurya *et al.* (1999 ▶). For the medical applications of ammonium picrate, see: Boericke (1982 ▶) and of morpholine derivatives, see: Lutz *et al.* (1947 ▶); Hazard *et al.* (1948 ▶); Raymond *et al.* (1999 ▶). For a related structure, see: Briggs *et al.* (2004 ▶). For a description of the Cambridge Structural Database, see: Allen (2002 ▶).
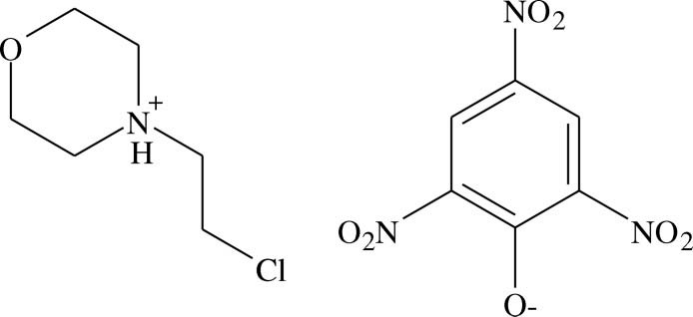


## Experimental

### 

#### Crystal data

C_6_H_13_ClNO^+^·C_6_H_2_N_3_O_7_^−^
*M* *_r_* = 378.72
Triclinic, 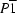

*a* = 8.2063 (4) Å
*b* = 9.3075 (5) Å
*c* = 10.2896 (6) Å
α = 93.954 (5)°
β = 95.284 (5)°
γ = 90.376 (4)°
*V* = 780.65 (7) Å^3^
*Z* = 2
Mo *K*α radiation
μ = 0.30 mm^−1^
*T* = 293 K
0.30 × 0.24 × 0.18 mm

#### Data collection

Oxford Diffraction Xcalibur diffractometer
Absorption correction: none
8478 measured reflections
4960 independent reflections
3665 reflections with *I* > 2σ(*I*)
*R*_int_ = 0.016

#### Refinement

*R*[*F* ^2^ > 2σ(*F* ^2^)] = 0.058
*wR*(*F* ^2^) = 0.179
*S* = 1.12
4960 reflections
286 parameters
H atoms treated by a mixture of independent and constrained refinement
Δρ_max_ = 0.85 e Å^−3^
Δρ_min_ = −0.61 e Å^−3^

### 

Data collection: *CrysAlis Pro* (Oxford Diffraction, 2007 ▶); cell refinement: *CrysAlis Pro*; data reduction: *CrysAlis RED* (Oxford Diffraction, 2007 ▶); program(s) used to solve structure: *SHELXS86* (Sheldrick, 2008 ▶); program(s) used to refine structure: *SHELXL97* (Sheldrick, 2008 ▶); molecular graphics: *ORTEP-3 for Windows*(Farrugia, 1997 ▶); software used to prepare material for publication: *WinGX* (Farrugia, 1999 ▶).

## Supplementary Material

Crystal structure: contains datablocks global, I. DOI: 10.1107/S1600536809035405/jh2096sup1.cif

Structure factors: contains datablocks I. DOI: 10.1107/S1600536809035405/jh2096Isup2.hkl

Additional supplementary materials:  crystallographic information; 3D view; checkCIF report

## Figures and Tables

**Table 1:** Hydrogen-bond geometry (Å, °)

*D*—H⋯*A*	*D*—H	H⋯*A*	*D*⋯*A*	*D*—H⋯*A*
N4—H4′⋯O1	0.91 (3)	1.89 (3)	2.718 (2)	151.2 (19)
N4—H4′⋯O2	0.91 (3)	2.25 (2)	2.896 (2)	127.6 (17)
C2—H2⋯O3	0.83 (3)	2.34 (3)	2.637 (3)	102 (2)
C2—H2⋯O4	0.83 (2)	2.45 (2)	2.723 (3)	101 (2)
C4—H4⋯O6	0.90 (3)	2.34 (3)	2.667 (3)	101.7 (15)
C7—H7*A*⋯O7^i^	0.93 (3)	2.54 (3)	3.026 (3)	113 (2)
C8—H8*B*⋯O5^ii^	0.94 (3)	2.47 (2)	3.290 (2)	146 (2)
C9—H9*A*⋯O5^ii^	0.97 (3)	2.45 (3)	3.341 (2)	154 (2)
C9—H9*B*⋯O2	0.97 (3)	2.34 (3)	2.985 (3)	123 (2)
C12—H12*A*⋯O1	0.96 (3)	2.57 (2)	3.254 (3)	129 (2)

Symmetry codes: (i) 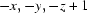; (ii) 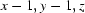.

**Fig. 1.**
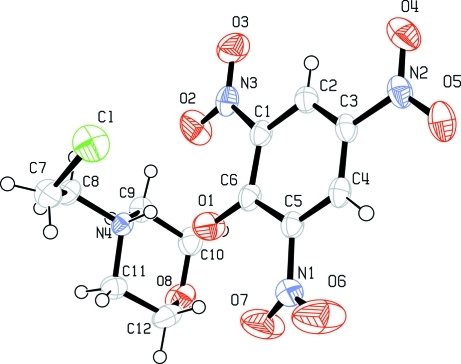
View of (I) (50% probability displacement ellipsoids).

**Fig. 2.**
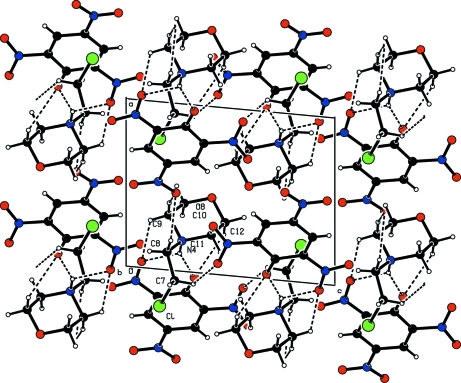
Depiction of C—H···O and N—H···O interactions in the title compound(I)

## supplementary crystallographic information

### Comment

The metal derivatives of picric acid are also known as medicines in homeopathy (Maurya *et al.*, 1999). Silver picrate is a good antimicrobial agent. Zinc picrate is used in Bright's disease, headache, exhaustion, facial paralysis, nymphomania, paralysis, seminal emissions, spiral weakness, loss of memory and energy.

Ammonium picrate is a remedy for malarial fever and neuralgia, whooping cough, pain in the occiput and mastoid regions (Boericke, 1982).

N-substituted morpholines were used as anti-malarials (Lutz *et al.*, 1947) and 4-methyl-4-(2-phenylethyl)-morpholinium iodide have shown marked hypertension in dogs at a dose of 5 mg./kg (Hazard *et al.*, 1948). Morpholine derivatives are also used as agricultural fungicides in cereals and are known as ergosterol biosynthesis inhibitors (Raymond *et al.*, 1999).

A search of the Cambridge Structural Database (Version 5.26; Allen, 2002) reveals that there are 90 known structures that contain the morpholinium cation. Of these there are 24 that have an *N*-ethyl chain, or longer, including the structure of 4-(2-fluoroethyl) morpholinium chloride (Briggs *et al.*, 2004).

The title compound, [C_6 _H_13_Cl N O]^+ ^[C_6_ H_2_N_3_ O_7_]^-^, was synthesized from picric acid and 4-(2-chloroethyl)morpholine.All geometrical parameters are in their usual ranges. The crystal structure is stabilized by C—H···O and N—H···O hydrogen interactions.

### Experimental

Picric acid (2.29 g, 0.01 mol) was dissolved in water. 4-(2-chloroethyl) morpholine (1.49 g 0.01 mol) was dissolved in 25 ml of ethanol. The two solutions were mixed and 5 ml of 5 *M* HCl was added to this mixture and stirred for few minutes,filtered, dried and yellow crystals of 4-(2-Chloroethyl)morpholinium picrate were obtained by slow evaporation in ethanol. (m.p. 394 K). Analytical data: Found (Cald): C %: 36.89(37.96); H%: 4.22 (4.25); N%:14.67(14.75).

### Refinement

All hydrogen atoms were located from the difference Fourier map and refined isotropically with distance restraints 0.830–0.982 Å.

### Crystal data

**Table:**

C_6_H_13_ClNO^+^·C_6_H_2_N_3_O_7_^−^	*Z* = 2
*M**_r_* = 378.72	*F*(000) = 392
Triclinic, *P*1	*D*_x_ = 1.611 Mg m^−^^3^
*a* = 8.2063 (4) Å	Mo *K*α radiation, λ = 0.71073 Å
*b* = 9.3075 (5) Å	Cell parameters from 3665 reflections
*c* = 10.2896 (6) Å	θ = 3.3–32.3°
α = 93.954 (5)°	µ = 0.30 mm^−^^1^
β = 95.284 (5)°	*T* = 293 K
γ = 90.376 (4)°	Rectangular, yellow
*V* = 780.65 (7) Å^3^	0.30 × 0.24 × 0.18 mm

### Data collection

**Table:**

Oxford Diffraction Xcalibur diffractometer	*R*_int_ = 0.016
ω–2θ scans	θ_max_ = 32.3°, θ_min_ = 3.3°
8478 measured reflections	*h* = −12→12
4960 independent reflections	*k* = −8→13
3665 reflections with *I* > 2σ(*I*)	*l* = −14→15

### Refinement

**Table:**

Refinement on *F*^2^	0 restraints
Least-squares matrix: full	0 constraints
*R*[*F*^2^ > 2σ(*F*^2^)] = 0.058	H atoms treated by a mixture of independent and constrained refinement
*wR*(*F*^2^) = 0.179	*w* = 1/[σ^2^(*F*_o_^2^) + (0.0877*P*)^2^ + 0.2886*P*] where *P* = (*F*_o_^2^ + 2*F*_c_^2^)/3
*S* = 1.12	(Δ/σ)_max_ < 0.001
4960 reflections	Δρ_max_ = 0.85 e Å^−^^3^
286 parameters	Δρ_min_ = −0.60 e Å^−^^3^

### Special details

**Table:**

Geometry. All e.s.d.'s (except the e.s.d. in the dihedral angle between two l.s. planes) are estimated using the full covariance matrix. The cell e.s.d.'s are taken into account individually in the estimation of e.s.d.'s in distances, angles and torsion angles; correlations between e.s.d.'s in cell parameters are only used when they are defined by crystal symmetry. An approximate (isotropic) treatment of cell e.s.d.'s is used for estimating e.s.d.'s involving l.s. planes.

### Fractional atomic coordinates and isotropic or equivalent isotropic displacement parameters (Å^2^)

**Table:**

	*x*	*y*	*z*	*U*_iso_*/*U*_eq_	
H8B	−0.178 (3)	−0.150 (3)	0.786 (2)	0.035 (6)*	
H9B	−0.286 (3)	0.146 (3)	0.883 (3)	0.041 (6)*	
H4	0.386 (3)	0.587 (2)	0.610 (2)	0.033 (6)*	
H9A	−0.401 (3)	0.024 (3)	0.793 (2)	0.036 (6)*	
H10A	−0.331 (3)	0.311 (3)	0.731 (2)	0.039 (6)*	
H2	0.299 (3)	0.541 (3)	0.972 (3)	0.042 (7)*	
H11A	−0.135 (3)	−0.023 (3)	0.548 (2)	0.040 (6)*	
H12A	−0.241 (3)	0.215 (3)	0.532 (3)	0.049 (7)*	
H8A	−0.102 (3)	−0.044 (2)	0.887 (3)	0.033 (6)*	
H11B	−0.300 (3)	−0.076 (3)	0.597 (2)	0.037 (6)*	
H7B	0.087 (4)	−0.208 (3)	0.791 (3)	0.058 (8)*	
H12B	−0.355 (4)	0.101 (3)	0.435 (3)	0.055 (8)*	
H7A	0.069 (3)	−0.121 (3)	0.678 (3)	0.052 (7)*	
H10B	−0.504 (4)	0.259 (3)	0.757 (3)	0.052 (7)*	
H4'	−0.106 (3)	0.125 (3)	0.743 (2)	0.037 (6)*	
Cl	0.22010 (6)	0.00390 (7)	0.83918 (7)	0.0572 (2)	
N4	−0.18045 (17)	0.05189 (15)	0.73274 (15)	0.0277 (3)	
C8	−0.1068 (2)	−0.0703 (2)	0.8042 (2)	0.0333 (4)	
C9	−0.3282 (2)	0.1067 (2)	0.7962 (2)	0.0343 (4)	
O8	−0.4535 (2)	0.1794 (2)	0.58892 (18)	0.0535 (4)	
C11	−0.2318 (2)	0.0085 (2)	0.5915 (2)	0.0365 (4)	
C10	−0.4054 (3)	0.2269 (2)	0.7217 (3)	0.0469 (5)	
C7	0.0592 (2)	−0.1150 (2)	0.7678 (2)	0.0405 (4)	
C12	−0.3138 (3)	0.1338 (3)	0.5261 (2)	0.0465 (5)	
C5	0.2198 (2)	0.43814 (18)	0.62764 (19)	0.0308 (3)	
N2	0.47503 (19)	0.70058 (17)	0.84556 (19)	0.0374 (4)	
C2	0.2800 (2)	0.51646 (19)	0.8933 (2)	0.0325 (4)	
O1	0.0317 (2)	0.25764 (19)	0.67334 (16)	0.0543 (5)	
C4	0.3325 (2)	0.54510 (18)	0.6696 (2)	0.0318 (4)	
C3	0.3613 (2)	0.58362 (18)	0.80201 (19)	0.0310 (4)	
C1	0.1667 (2)	0.40844 (18)	0.85050 (19)	0.0311 (4)	
C6	0.1275 (2)	0.35914 (19)	0.71437 (19)	0.0330 (4)	
N3	0.0845 (2)	0.34768 (17)	0.95407 (18)	0.0384 (4)	
N1	0.1924 (2)	0.40692 (18)	0.48624 (17)	0.0389 (4)	
O5	0.5335 (2)	0.76658 (17)	0.76105 (19)	0.0541 (4)	
O2	−0.0347 (2)	0.2682 (2)	0.92512 (19)	0.0603 (5)	
O4	0.5057 (2)	0.73018 (19)	0.96342 (19)	0.0542 (4)	
O3	0.1342 (2)	0.3801 (2)	1.06775 (18)	0.0568 (5)	
O7	0.0693 (3)	0.3444 (3)	0.4397 (2)	0.0808 (7)	
O6	0.2896 (3)	0.4530 (3)	0.4174 (2)	0.0902 (8)	

### Atomic displacement parameters (Å^2^)

**Table:**

	*U*^11^	*U*^22^	*U*^33^	*U*^12^	*U*^13^	*U*^23^
Cl	0.0304 (2)	0.0661 (4)	0.0743 (5)	−0.0029 (2)	0.0023 (2)	0.0011 (3)
N4	0.0240 (6)	0.0286 (6)	0.0314 (7)	−0.0047 (5)	0.0033 (5)	0.0069 (5)
C8	0.0287 (8)	0.0346 (9)	0.0378 (10)	−0.0009 (7)	0.0032 (7)	0.0116 (7)
C9	0.0269 (8)	0.0356 (9)	0.0411 (10)	−0.0010 (6)	0.0065 (7)	0.0041 (7)
O8	0.0401 (8)	0.0624 (10)	0.0567 (11)	0.0076 (7)	−0.0100 (7)	0.0140 (8)
C11	0.0333 (9)	0.0426 (10)	0.0330 (9)	−0.0036 (7)	0.0009 (7)	0.0027 (7)
C10	0.0431 (11)	0.0402 (10)	0.0573 (14)	0.0088 (9)	0.0011 (10)	0.0067 (9)
C7	0.0340 (9)	0.0425 (10)	0.0449 (12)	0.0056 (8)	0.0033 (8)	0.0012 (9)
C12	0.0446 (11)	0.0585 (13)	0.0362 (11)	−0.0044 (10)	−0.0058 (9)	0.0152 (9)
C5	0.0287 (7)	0.0271 (7)	0.0367 (9)	−0.0030 (6)	0.0012 (6)	0.0057 (6)
N2	0.0289 (7)	0.0288 (7)	0.0537 (11)	−0.0048 (6)	0.0011 (7)	0.0009 (7)
C2	0.0306 (8)	0.0285 (8)	0.0382 (10)	−0.0009 (6)	0.0009 (7)	0.0042 (7)
O1	0.0577 (10)	0.0596 (10)	0.0445 (9)	−0.0371 (8)	−0.0041 (7)	0.0103 (7)
C4	0.0265 (7)	0.0258 (7)	0.0442 (10)	−0.0024 (6)	0.0056 (7)	0.0089 (7)
C3	0.0261 (7)	0.0242 (7)	0.0425 (10)	−0.0044 (6)	0.0011 (7)	0.0036 (7)
C1	0.0293 (8)	0.0284 (7)	0.0366 (9)	−0.0038 (6)	0.0041 (7)	0.0083 (7)
C6	0.0282 (8)	0.0305 (8)	0.0409 (10)	−0.0069 (6)	0.0002 (7)	0.0103 (7)
N3	0.0397 (8)	0.0313 (7)	0.0467 (10)	−0.0014 (6)	0.0122 (7)	0.0101 (7)
N1	0.0435 (9)	0.0361 (8)	0.0369 (9)	−0.0018 (7)	0.0037 (7)	0.0020 (7)
O5	0.0504 (9)	0.0417 (8)	0.0711 (12)	−0.0223 (7)	0.0093 (8)	0.0074 (8)
O2	0.0626 (11)	0.0575 (10)	0.0627 (11)	−0.0316 (8)	0.0238 (9)	−0.0016 (8)
O4	0.0494 (9)	0.0505 (9)	0.0590 (11)	−0.0126 (7)	−0.0051 (8)	−0.0080 (8)
O3	0.0639 (11)	0.0647 (11)	0.0439 (9)	−0.0126 (9)	0.0067 (8)	0.0163 (8)
O7	0.0923 (16)	0.1001 (17)	0.0461 (11)	−0.0532 (14)	−0.0013 (10)	−0.0075 (10)
O6	0.0833 (15)	0.142 (2)	0.0462 (11)	−0.0453 (16)	0.0161 (10)	0.0066 (13)

### Geometric parameters (Å, °)

**Table:**

Cl—C7	1.788 (2)	C12—H12A	0.96 (3)
N4—C8	1.497 (2)	C12—H12B	0.99 (3)
N4—C11	1.503 (2)	C5—C4	1.374 (2)
N4—C9	1.504 (2)	C5—C6	1.455 (2)
N4—H4'	0.91 (2)	C5—N1	1.460 (3)
C8—C7	1.500 (3)	N2—O4	1.228 (3)
C8—H8B	0.94 (2)	N2—O5	1.229 (2)
C8—H8A	0.87 (3)	N2—C3	1.449 (2)
C9—C10	1.510 (3)	C2—C3	1.382 (3)
C9—H9B	0.98 (3)	C2—C1	1.386 (2)
C9—H9A	0.97 (2)	C2—H2	0.83 (3)
O8—C12	1.420 (3)	O1—C6	1.250 (2)
O8—C10	1.426 (3)	C4—C3	1.383 (3)
C11—C12	1.517 (3)	C4—H4	0.89 (2)
C11—H11A	0.98 (3)	C1—C6	1.450 (3)
C11—H11B	0.97 (2)	C1—N3	1.456 (2)
C10—H10A	0.98 (3)	N3—O3	1.220 (2)
C10—H10B	0.96 (3)	N3—O2	1.225 (2)
C7—H7B	0.93 (3)	N1—O7	1.206 (3)
C7—H7A	0.94 (3)	N1—O6	1.210 (3)
			
C8—N4—C11	112.20 (15)	Cl—C7—H7A	105.7 (17)
C8—N4—C9	110.07 (14)	H7B—C7—H7A	103 (3)
C11—N4—C9	108.38 (14)	O8—C12—C11	111.16 (18)
C8—N4—H4'	106.9 (15)	O8—C12—H12A	106.5 (17)
C11—N4—H4'	113.0 (15)	C11—C12—H12A	110.6 (16)
C9—N4—H4'	106.1 (15)	O8—C12—H12B	106.1 (17)
N4—C8—C7	115.12 (16)	C11—C12—H12B	108.8 (16)
N4—C8—H8B	107.4 (14)	H12A—C12—H12B	113 (2)
C7—C8—H8B	108.0 (14)	C4—C5—C6	124.17 (18)
N4—C8—H8A	106.8 (16)	C4—C5—N1	116.02 (16)
C7—C8—H8A	110.2 (15)	C6—C5—N1	119.80 (15)
H8B—C8—H8A	109 (2)	O4—N2—O5	123.60 (17)
N4—C9—C10	110.06 (17)	O4—N2—C3	118.95 (17)
N4—C9—H9B	105.2 (15)	O5—N2—C3	117.44 (18)
C10—C9—H9B	108.9 (15)	C3—C2—C1	118.96 (18)
N4—C9—H9A	105.1 (14)	C3—C2—H2	120.0 (18)
C10—C9—H9A	111.6 (14)	C1—C2—H2	121.0 (18)
H9B—C9—H9A	116 (2)	C5—C4—C3	119.30 (16)
C12—O8—C10	109.70 (17)	C5—C4—H4	118.7 (15)
N4—C11—C12	109.86 (17)	C3—C4—H4	122.0 (15)
N4—C11—H11A	109.0 (14)	C2—C3—C4	121.53 (16)
C12—C11—H11A	111.4 (14)	C2—C3—N2	119.26 (17)
N4—C11—H11B	102.7 (14)	C4—C3—N2	119.18 (16)
C12—C11—H11B	116.2 (14)	C2—C1—C6	124.15 (16)
H11A—C11—H11B	107 (2)	C2—C1—N3	114.57 (17)
O8—C10—C9	111.16 (18)	C6—C1—N3	121.27 (15)
O8—C10—H10A	113.2 (15)	O1—C6—C1	125.59 (17)
C9—C10—H10A	109.7 (14)	O1—C6—C5	122.46 (18)
O8—C10—H10B	105.5 (17)	C1—C6—C5	111.90 (15)
C9—C10—H10B	111.4 (17)	O3—N3—O2	121.69 (18)
H10A—C10—H10B	106 (2)	O3—N3—C1	118.95 (17)
C8—C7—Cl	113.24 (15)	O2—N3—C1	119.35 (18)
C8—C7—H7B	113.2 (19)	O7—N1—O6	121.2 (2)
Cl—C7—H7B	107.2 (18)	O7—N1—C5	119.83 (18)
C8—C7—H7A	114.0 (18)	O6—N1—C5	118.78 (18)

### Hydrogen-bond geometry (Å, °)

**Table:**

*D*—H···*A*	*D*—H	H···*A*	*D*···*A*	*D*—H···*A*
N4—H4'···O1	0.91 (3)	1.89 (3)	2.718 (2)	151.2 (19)
N4—H4'···O2	0.91 (3)	2.25 (2)	2.896 (2)	127.6 (17)
C2—H2···O3	0.83 (3)	2.34 (3)	2.637 (3)	102 (2)
C2—H2···O4	0.83 (2)	2.45 (2)	2.723 (3)	101 (2)
C4—H4···O6	0.90 (3)	2.34 (3)	2.667 (3)	101.7 (15)
C7—H7A···O7^i^	0.93 (3)	2.54 (3)	3.026 (3)	113 (2)
C8—H8B···O5^ii^	0.94 (3)	2.47 (2)	3.290 (2)	146 (2)
C9—H9A···O5^ii^	0.97 (3)	2.45 (3)	3.341 (2)	154 (2)
C9—H9B···O2	0.97 (3)	2.34 (3)	2.985 (3)	123 (2)
C12—H12A···O1	0.96 (3)	2.57 (2)	3.254 (3)	129 (2)

Symmetry codes: (i) −*x*, −*y*, −*z*+1; (ii) *x*−1, *y*−1, *z*.
